# An intein with genetically selectable markers provides a new approach to internally label proteins with GFP

**DOI:** 10.1186/1472-6750-11-71

**Published:** 2011-06-27

**Authors:** Richard Ramsden, Luther Arms, Trisha N Davis, Eric GD Muller

**Affiliations:** 1 Department of Biochemistry, University of Washington, Seattle, WA, USA

## Abstract

**Background:**

Inteins are proteins that catalyze their own removal from within larger precursor proteins. In the process they splice the flanking protein sequences, termed the N-and C-terminal exteins. Large inteins frequently have a homing endonuclease that is involved in maintaining the intein in the host. Splicing and nuclease activity are independent and distinct domains in the folded structure. We show here that other biochemical activities can be incorporated into an intein in place of the endonuclease without affecting splicing and that these activities can provide genetic selection for the intein. We have coupled such a genetically marked intein with GFP as the N-terminal extein to create a cassette to introduce GFP within the interior of a targeted protein.

**Results:**

The *Pch *PRP8 mini-intein of *Penicillium chrysogenum *was modified to include: 1) aminoglycoside phosphotransferase; 2) imidazoleglycerol-phosphate dehydratase, His5 from *S. pombe *; 3) hygromycin B phosphotransferase; and 4) the transcriptional activator LexA-VP16. The proteins were inserted at the site of the lost endonuclease. When expressed in *E. coli*, all of the modified inteins spliced at high efficiency. Splicing efficiency was also greater than 96% when expressed from a plasmid in *S. cerevisiae*. In addition the inteins conferred either G418 or hygromycin resistance, or histidine or leucine prototropy, depending on the inserted marker and the yeast genetic background. DNA encoding the marked inteins coupled to GFP as the N-terminal extein was PCR amplified with ends homologous to an internal site in the yeast calmodulin gene *CMD1*. The DNA was transformed into yeast and integrants obtained by direct selection for the intein's marker. The His5-marked intein yielded a fully functional calmodulin that was tagged with GFP within its central linker.

**Conclusions:**

Inteins continue to show their flexibility as tools in molecular biology. The *Pch *PRP8 intein can successfully tolerate a variety of genetic markers and still retain high splicing efficiency. We have shown that a genetically marked intein can be used to insert GFP in one-step within a target protein *in vivo*.

## Background

Inteins are small proteins naturally found either embedded within a larger precursor protein (cis-acting inteins), or split between two pro-proteins (trans-acting inteins). They have the unique ability to self-catalyze their excision from the pro-protein(s) and in the process form a peptide bond between the flanking amino acid sequences that reside at the N-and C-terminal boundaries with the intein [1,2]. These flanking regions adjacent to the intein are referred to as exteins, analogous to the terminology for introns and exons. An intein acts autonomously, splicing together the exteins without the need of a co-factor or an assisting complex. Since splicing is post-translational, for cis-acting inteins a single open reading frame driven by a single promoter expresses two distinct mature protein products: the spliced exteins and the intein.

The first intein described, *Sce *VMA1, was found in a subunit of the vacuolar H^+^-adenosine triphosphatase complex of the budding yeast *Saccharomyces cerevisiae *[3,4]. Currently over 500 inteins are known, found in over 200 species of microorganisms in over 70 different proteins [5]. Present in all three phylogenetic domains of life, inteins in Eukaryotes are predominantly found in fungi and unicellular alga [5].

A homing endonuclease is often present within cis-acting inteins. The splicing and endonuclease domains are structurally and functionally distinct, as shown both by the crystal structure of *Sce *VMA1 [6] and the conservation of splicing following the deletion of the homing endonuclease [1,7]. In addition minimal or mini-inteins lacking the homing endonuclease occur naturally or have been engineered to be as small as 143 amino acids [8,9].

The use of inteins in molecular biology, biotechnology and drug discovery has recently been reviewed [10-12] . The broad spectrum of applications for inteins is in part due to the minimal constraints placed on the sequence of the exteins. The only absolute requirement is the presence of cysteine, serine or threonine at the downstream splice junction in the C-terminal extein. There are no known size constraints. Splicing efficiency is reduced if the extein domains are unfolded or have poor solubility, but *in vitro *even this can usually be overcome with detergents or refolding regimes [13]. The immediate flanking sequence of the intein can affect splicing efficiency to varying degrees [14], but a variety of sequences are tolerated. Thus the inherent tolerance of inteins allows them to be adapted to splice proteins of diverse character.

In this study the flexibility of inteins has been exploited to investigate the potential for inteins to direct *in vivo *gene modification, specifically the tagging of an internal region of a protein with GFP. The microscopy group of the Yeast Resource Center is focused on using Förster resonance energy transfer, FRET, to study the organization of large protein complexes *in vivo *[15-18]. A limiting step for FRET studies is identifying regions within proteins that can accept a GFP-related fluorescent protein tag. As a first step towards overcoming this limitation, we engineered genetic markers within the *Pch *PRP8 intein that provided a way to internally tag proteins in one step in yeast. The P*ch *PRP8 intein was chosen for its robust, temperature-independent splicing activity [9,19] and the engineered inteins continued to display very high splicing efficiency.

## Results

### *Pch *PRP8 intein splices with inserts at the empty endonuclease site

During the course of their initial characterization of the *Pch *PRP8 mini-intein Elleuche *et al. *[19] aligned the sequence of the intein with PRP8 inteins from other species. The position of a presumptive lost homing endonuclease was identified from a gap in the alignment. This position, designated "E", later was shown to tolerate a deletion of six amino acids without loss of splicing activity [9]. In addition the intein could be split in half at this position and the resulting two independent proteins, when expressed in *E. coli*, had active *trans*-splicing activity [20].

**Figure 1:**
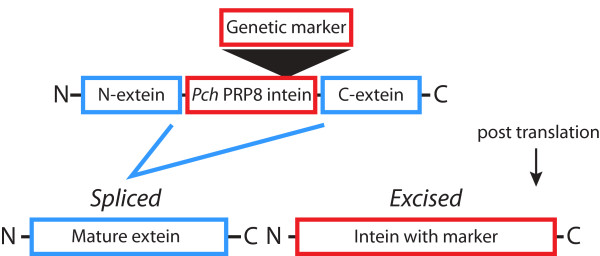
**A schematic of genetic selection using marked *Pch *PRP8 inteins**. N-and C-terminal exteins, boxed in blue, flank the intein, boxed in red, as shown. Following translation the intein catalyzes the ligation of the exteins and the *Pch *PRP8 marker-intein is released. Markers used for genetic selection were inserted between amino acid positions 114 and 115 of the intein.

Given the tolerance of the "E" position for change, this site in the *Pch *PRP8 intein was chosen for the insertion of genes encoding genetically selectable enzymes. These enzymes were: 1) aminoglycoside phosphotransferase; 2) imidazoleglycerol-phosphate dehydratase from *S. pombe*; and 3) hygromycin B phosphotransferase. They confer G418/kanamycin resistance, histidine prototrophy and hygromycin resistance in the appropriate yeast genetic background, respectively. In addition the gene encoding the transcriptional activator LexA-VP16 [21] was inserted. These proteins share in common a modest size (216, 269, 280 and 341 amino acids for His5, G418^R^, LexA-VP16 and Hyg^R^, respectively) all below the typical size of homing endonucleases (350-450 aa). Perhaps more importantly the N-and C-terminal ends of each protein are proximal to each other [22-25]. A schematic diagram of these constructs is shown in Figure 1.

We used the experimental system of Elleuche *et al. *[19] to test whether the *Pch *PRP8 intein with inserted markers could splice. In their system GST is the N-terminal extein and a 6 × His-tag is the C-terminal extein. The embedded intein is expressed in *E. coli *and splicing results in the production of GST-6 × His and the released intein.

**Figure 2:**
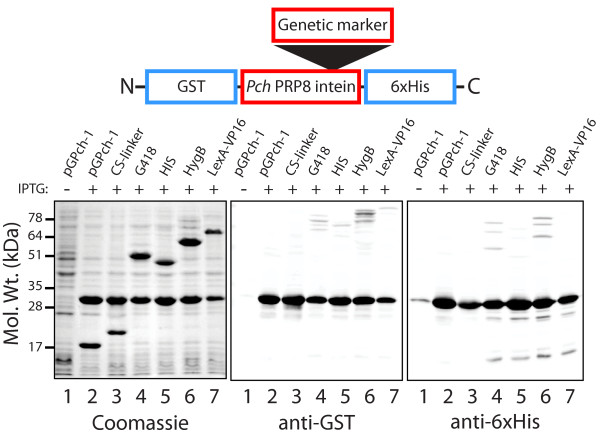
**The marked *Pch *PRP8 inteins splice at high efficiency when expressed in *E. coli***. SDS-PAGE and Western blots of whole cell lysates of *E. coli *carrying plasmids expressing the modified inteins. The N-and C-terminal exteins are GST (29 kD) and 6 × His (3 kD), respectively. The markers inserted within the intein are indicated above each lane in the gel. Plasmid pGPch-1 is the starting vector with an unmodified *Pch *PRP8 intein, and CS-linker refers to pGPch-1 with a cloning site and linkers. The predicted molecular weights (kDa) of the inteins are: *Pch *PRP8 (18), *Pch *PRP8-cloning site + linker (19); *Pch *PRP8-G418^R ^(49); *Pch *PRP8-His5 (42); *Pch *PRP8-HygB^R ^(57); *Pch *PRP8-LexA-VP16 (51). *Pch *PRP8-LexA-VP16 migrates anomalously. GST-6 × His is ~30 kD. Expression of the inteins was driven by a tac promoter and was induced in lanes 2-7 with 0.2 mM IPTG. Western blot analysis was performed as described in Methods. The strong band at ~30 kD is the spliced extein, GST-6 × His. Plasmids in the host *E. coli *strain: Lanes 1 and 2, pGPch-1; Lane 3, pRR01; Lane 4, pRR02; Lane 5, pRR03; Lane 6, pRR04; Lane 7, pRR05.

We confirmed the robust splicing activity of *Pch *PRP8 intein (Figure 2, lane 2). GST-6 × His and the released intein are clearly visible in SDS-PAGE, and the identity of spliced GST-6 × His was confirmed by Western blot analysis with anti-6 × His and anti-GST antibodies. The addition of cloning sites flanked by flexible protein linkers at position E did not impair splicing since the free inteins and the GST-6 × His were clearly visible in SDS-PAGE with no evidence of unspliced products (Figure 2, lane 3).

The modified inteins with the 4 different selection marker cassettes also showed highly efficient splicing (Figure 2, lanes 4-7). Based on the ratio of spliced products to larger, unspliced proteins in the Western blots, the splicing efficiencies ranged from ~88% for the *Pch *PRP8 Hyg^R^-intein to almost 100% for the His5-and LexA-VP16-inteins. Thus the *Pch *PRP8 intein tolerated the insertion of four different proteins at the vacant endonuclease site, maintaining nearly complete excision and splicing activity in this system.

### The marked *Pch *PRP8 inteins splice in yeast and confer selectable genetic traits

To determine whether the modified inteins would function in yeast we constructed a new panel of yeast *CEN4/URA3 *plasmids that contained the marked inteins expressed under the control of the *GAL1 *promoter. For these plasmids the N-terminal extein was GFP containing a nuclear localization signal (NLS) and the C-terminal extein was the FLAG peptide, DYKDDDDK. Just as in the previous *E. coli *plasmids, the extein/intein junction encodes five N-and four C-terminal extein residues of the *P. chrysogenum *Prp8 protein. The spliced product is NLS-GFP-FLAG (33 kD) and the released intein carries the selectable genetic marker or a transcription factor. NLS-GFP provided a simple way to follow expression and proper folding of the extein.

**Figure 3:**
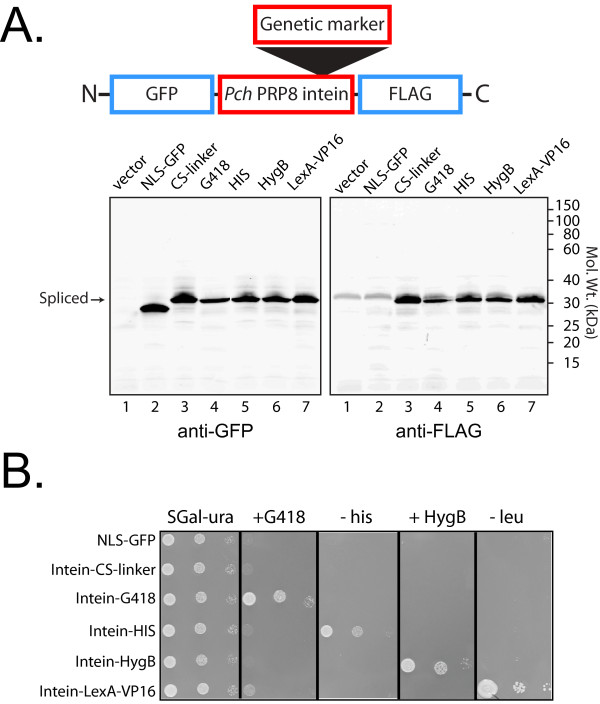
**Marked *Pch *PRP8 inteins splice and allow genetic selection in yeast**. The inteins described in Figure 2, but with different exteins, were expressed in yeast under the control of the *GAL1 *promoter. For expression in yeast, the N-extein was GFP with a nuclear localization signal, and the C-extein was a FLAG epitope tag. The spliced extein product is NLS-GFP-FLAG (33 kD). The molecular weights of the *Pch *marker-inteins are given in Figure 2. However in the anti-GFP and anti-FLAG Westerns there is no clear evidence for unspliced or partially spliced products of higher molecular weight than the spliced product, such as seen in Figure 2. **A**. Western blots showing the high efficiency of splicing in strain YL (2,4) LU expressing the modified inteins (and controls) from the following plasmids: Lane 1, pBM258; Lane 2, pRR26; Lane 3, pRR21; Lane 4, pRR22; Lane 5, pRR23; Lane 6, pRR24; Lane 7, pRR25. Analysis was performed as described in Methods. The genetic markers of the inteins and antibodies used for Western blots are indicated above and below the gels, respectively. Note that in the anti-FLAG western a background band was apparent in all lanes, including the protein extracts from the vector alone and NLS-GFP expression in lanes 1 and 2. **B**. Five-fold serial dilutions of the various yeast strains were prepared and spotted onto SGal-ura medium, SGal-ura medium lacking histidine or leucine, or SGal-ura supplemented with antibiotic as indicated. The plasmid backbone confers uracil prototrophy.

In agreement with the results in *E. coli*, all of the modified inteins when expressed in yeast displayed high efficiency splicing (Figure 3). Based on the limits of detection in the anti-GFP Westerns, we estimate that the efficiency of splicing is greater than 96% for the modified inteins.

Concomitant with splicing the intein is released from the precursor protein. The inteins did not inhibit growth and provided resistance to G418 or hygromycin in cells expressing the *Pch *PRP8 G418^R^-intein or the HygB^R^-intein, respectively (Figure 3B). Likewise the *Pch *PRP8 His5-intein was able to complement the mutation in *HIS3 *in this genetic background (Figure 3B). Finally the *Pch *PRP8 LexA-VP16-intein was able to drive expression of a *LEU2 *gene that had 2 *lexA *operator sequences within its promoter and confer selectable leucine prototrophy to the yeast strain. Thus in all cases each marker remained functional within the context of the excised intein.

### Direct integration of the marked inteins at the *CMD1 *locus

**Figure 4:**
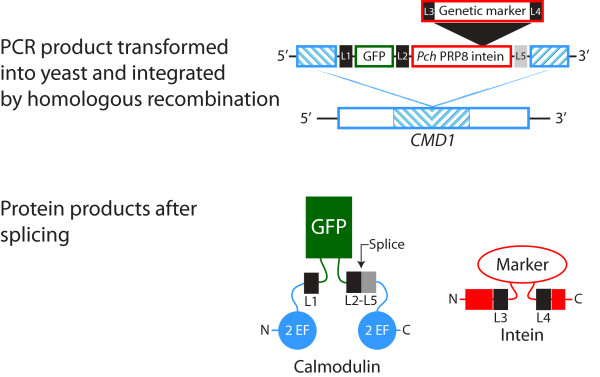
**Schematic showing the experimental design to integrate the marked *Pch *PRP8 inteins at the *CMD1 *locus**. The top panel shows the composition and organization of the PCR amplified DNA used to transform yeast. The blue hatched area corresponds to the central sequence of the *CMD1 *gene that was the target for homologous recombination. L1-L5 are linker regions. In the bottom panel are the two proteins produced after splicing: 1) calmodulin with GFP inserted between S79 and N80, and 2) *Pch *PRP8 intein with genetic markers inserted at the vacant endonuclease site. The junction between L2 and L5 is the splice site. L2 and L5 contain 5 and 4 amino acids from the original *Pch *Prp8 extein sequences. For a description of the linker sequences see Methods. EF refers to the calcium-binding EF-hand domains. The sizes of the regions are drawn for clarity and are not to scale.

Having established that the intein remained active and the embedded markers functional in yeast, we next examined whether the marked inteins could provide a method to introduce GFP within the coding sequence of a targeted gene. For our test we chose *CMD1*, the gene encoding yeast calmodulin [26]. Calmodulin contains two globular EF-hand calcium-binding domains separated by a flexible linker. The design of the experiment is presented in Figure 4. Briefly the GFP-*Pch *PRP8 marker-intein cassette was PCR amplified using oligonucleotide primers with ends homologous to the center of the *CMD1 *gene encoding the central linker region. Transformation and direct selection for the markers within the inteins isolates cells in which the *Pch *PRP8 *GFP*-intein cassettes were inserted by homologous recombination between amino acids 79 and 80 of calmodulin.

Transformation with DNA from the *Pch *PRP8 G418^R^-, His5-and HygB^R^-inteins produced transformants with the selected auxotrophic or antibiotic resistance, while the *Pch *PRP8 LexA-VP16-intein did not yield transformants. Since the level of expression of the intein is dependent on the level of *CMD1 *expression we conclude that the LexA-VP16 was not at a level to fully activate *lexAop_6_-LEU2 *gene expression in strain YL [6,4]LU. However in agreement with the previous results from yeast episomal expression, the recovery of resistant or prototrophic cells from the other *Pch *PRP8 marked-inteins again showed that the intein domain did not impair the activity of the selection markers.

**Figure 5:**
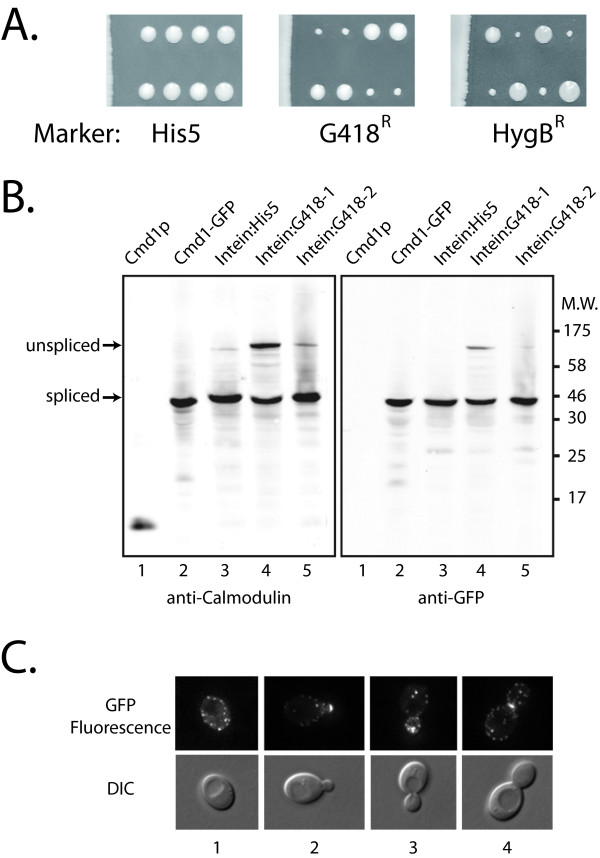
**Integration of the marked *Pch *PRP8 inteins at *CMD1 *locus**. **A**. Tetrad analysis of diploids that are heterozygous for the insertion of the marked inteins at the *CMD1 *locus (*CMD1/CMD1::GFP-Pch PRP8 marker-intein*). Individual haploid spores were grown on non-selective YPD medium. Segregation of the mutant alleles was 2:2 and the slow growing colonies contain *CMD1 *with the G418^R^-and HygB^R^-intein insertions as determined by plating the haploids on selective medium (not shown). **B**. Western blot analysis of protein extracts from haploids containing the *Pch *PRP8 marked-inteins at the *CMD1 *locus. Lane 1, 100 ng purified calmodulin; Lane 2 LAY18-2C expressing Cmd1-GFP (constructed using the classic PCR method [41]); Lane 3, extract from a haploid *CMD1::GFP-Pch PRP8-HIS5-intein *isolate; Lanes 4, 5 extracts from two separate haploid *CMD1::GFP-Pch PRP8-G418^R^-intein *isolates. **C**. Fluorescence microscopy of the haploid *CMD1::GFP-Pch PRP8-HIS5-intein *isolate. Numbers 1-4 refer to images showing unbudded, small budded, medium budded or large budded cells, respectively.

In our experimental design the intein was initially transformed into a diploid strain so that transformants could be recovered even if the integration of the intein impaired the expression or activity of calmodulin. Diploid transformants were sporulated and the tetrads dissected to yield four haploids, two with the intein insertion and two without. The results of the tetrad analysis are shown in Figure 5A. The *Pch *PRP8 His5-intein *CMD1 *insertions grew at the same rate as wild-type, but both the *Pch *PRP8 G418^R^-and HygB^R^-inteins impaired growth.

Associated with the impaired growth was evidence of inefficient splicing (Figure 5B). The *Pch *PRP8 His5-intein was effectively spliced from calmodulin, producing Cmd1-GFP at high efficiency (lane 3). On the other hand, the G418^R^-intein (lanes 4,5) and HygB^R^-intein (not shown) were not. Different isolates showed varying levels of accumulation of unspliced product (lanes 4 and 5). The slow growth and phenotypic variation of the G418^R ^and Hyg^R ^isolates precluded further analysis and in any case meant that these inteins were not suitable for tagging Cmd1.

Calmodulin concentrates at sites of cell growth and at the spindle pole body in yeast [27-29]. The wild-type localization pattern was seen for Cmd1 with the internal GFP-His5-intein (Figure 5C). In agreement with the published results, unbudded cells had calmodulin in puncta at the cell membrane. In newly budded cells calmodulin formed a patch at the bud tip that dispersed as the bud grew. Preceding cytokinesis calmodulin was concentrated at the bud neck. Localization at the pair of spindle pole bodies is best seen in the medium budded cell in Figure 5. For comparison the online Yeast Resource Center Public Image Repository [29] contains over 2000 images of yeast showing the localization of *CMD1 *tagged with different fluorescent proteins.

## Discussion

Inteins are widely used in biotechnology to manipulate proteins. The capacity of inteins to break and form peptide bonds has been exploited to develop a range of methodologies from a routine procedure to purify proteins, to more advanced semi-synthetic applications that assemble proteins for NMR, crystallography and mechanistic studies. These uses and others have been recently reviewed [10,11,30].

The use of inteins as genetic tools is still an emerging area of research. Temperature-sensitive inteins [31-33], light-activated inteins [34], and inteins controlled by small molecules [35-37], provide a way to control protein activity by controlling when a protein that has been interrupted by an intein is spliced together to yield its mature form. Conditional protein assembly by inteins *in vivo *is a promising method to generate temperature-sensitive mutants, and target when and where a protein is functional in a cell.

Here we add a new role for inteins in genetic manipulations, as a genetic marker with the unique property of self-excision. The engineered intein marks the expression of the protein into which it is embedded and yet after splicing is not a part of the mature protein. We demonstrate in yeast the use of a *Pch *PRP8 His5-marked intein to deliver, in one step and *in vivo*, GFP within a targeted protein without disturbing the function of the protein. In principle, the method could be extended to other protein tags, and adapted to other organisms.

We created a family of genetically marked inteins based on the PRP8 intein of *Penicillium chrysogenum*. PRP8 inteins have an ancient heritage and are widespread [19,38-40]. However they are not found in *S. cerevisiae*. The *Pch *PRP8 intein and the native yeast *VMA1 *intein are sufficiently divergent (21% sequence identity) to preclude homologous recombination between the two during the transformation step. For comparison the *S. cerevisiae HIS3 *and the *S. pombe his5^+ ^*genes share 58% sequence identity and yet do not recombine during integrative transformation [41]. The sequence divergence, the fact that the *Pch *PRP8 intein is well characterized [9,19,20,39], splices at high efficiency across a broad range of temperatures [19] and lacks a DNA binding domain led to its choice in our studies.

The primary sequence of the *Pch *PRP8 mini-intein shares sequence similarity with other inteins [20]. Thus its three-dimensional structure likely conforms to the canonical Hedgehog/Intein (HINT) protein scaffold [42-48]. The HINT module has a flat horseshoe shape with a series of β-strands forming the framework. The active-site is centered at the interior base or "toe" of the horseshoe. Endonucleases if present in the intein are a separate domain located away from the active site region at the "heel" of the horseshoe.

Here we show that the *Pch *PRP 8 intein, and presumably other inteins, have a remarkable capacity to accept foreign protein domains at the region of the structure where the homing endonuclease resides in full inteins. High splicing efficiency was observed for all of the inserted single domain proteins that were used for genetic markers. We attribute the success to several factors. An alignment of the *Pch *PRP8 intein with the *Synechocystis sp*. DnaB and the *Mycobacterium xenopi *GyrA inteins shows that the insertion site for our genetic markers is located in disordered loops in the crystal structures of both of these inteins [42,44]. Thus the region of insertion is inherently flexible and unstructured. In addition, one shared feature of the proteins used for markers is that their N-and C-termini are proximal [22-25]. The exit of the inserted protein domain out of the intein and its return occurs nearby in space and would tend to minimize strain in the structure of the intein. Since the majority of single domain proteins have some region of their two termini within 5 Å [49,50] and termini are consistently found at the surface of the protein [51], we predict that many single domain proteins could be inserted into inteins without detriment to splicing efficiency.

Three enzymes and a transcription factor were used as genetic markers to modify the *Pch *PRP8 intein. In pilot experiments all four modified inteins showed high splicing efficiency, both in *E. coli *and yeast, and in yeast the proteins within the excised inteins were active and enabled growth under conditions of selection. However when these inteins were used to deliver GFP within calmodulin only the strain tagged with the *Pch *PRP8 His5-intein grew normally. Under selective conditions transformation with the *Pch *PRP8 LexA-VP16-intein did not yield any transformants, and the G418^R^-and Hyg^R^-inteins yielded cells with poor growth and diminished splicing efficiency. The disruption of calmodulin function was surprising, given the pilot results, and is not understood. It is possible that the unspliced products which accumulate interfere with growth. We are actively investigating ways to boost the level of stable protein and the efficiency of splicing through changes in codon usage and the composition of the linkers between the various domains. In addition, given the success of the *Pch *PRP8 His5-intein, we are constructing other inteins with metabolic markers that may require lower levels of expression.

Our intein based method to insert the gene encoding GFP within an ORF offers several advantages over other PCR-based methods available in yeast. Other methods [52-54] introduce a gene with its own promoter that must be first be removed before the GFP is correctly in frame. Thus a second recombination event is required, either catalyzed by the induction of Cre-recombinase or by a rare natural recombination event that is isolated by genetic selection, to remove the genetic marker with its associated promoter. In addition the Cre-recombinase based system leaves behind within the ORF a peptide sequence coded for by the remaining *loxP *site, a sequence that can disrupt activity (unpublished). The introduced gene also temporarily interrupts the expression of the target protein. With the intein-based method integration and tagging occur in one step. Since the marked inteins are inserted in frame, expression of the target is only disrupted during recombination. Given these advantages marked inteins show great promise and complement other available methods.

## Conclusions

We have shown that the *Pch *PRP8 intein can incorporate a variety of selectable genetic markers and still exhibit high splicing activity. This led to the development of a simple method to insert GFP within the interior of a protein in yeast. This novel use of an intein has the potential to provide a way to add, substitute, or delete domains within targeted proteins in a single step in yeast. In addition it may find application in other systems as well, such as recombineering, a method that is also based on genetic manipulation through recombination.

Refinement of this method is underway at the Yeast Resource Center. Primary areas to investigate will be the effect of codon optimization, the utility of other selection markers, and cassette development for other useful tags such as the FRET partners YFP and CFP.

## Methods

### Strains and media

KGY315 [55] (*MATa/MATα, ade2-1oc/ade2-1oc, ADE3/ade3Δ100, can1-100/can1-100, CYH2^s^/cyh2^r^, his3-11,15/his3-11,15, leu2-3,112/leu2-3,112, trp1-1/trp1-1, ura3-1/ura3-1*) was derived from W303 [56]. YL(2, 4)LU (*MATa, his3, trp1, leu2::lexAop_2_-LEU2, ura3::lexAop_4_-SPO13*TATA-*URA3*) is a reporter strain for LexA-VP16 expression [21]. YL(6, 4)LU is the same as YL(2, 4)LU except that there are 6 *lexA*op sequences before *LEU2*. LAY18-2C was derived from KGY315 in which the C-terminus of *CMD1 *was tagged with GFP as described at the Yeast Resource Center web site http://depts.washington.edu/yeastrc/pages/fm_3.html. *Escherichia coli *strain Tuner™(DE3) (EMB, San Diego, CA) was used for expression and XL10-Gold^® ^(Stratagene, La Jolla, CA) for routine cloning.

Yeast were grown in either synthetic dextrose minimal media (SD) or synthetic galactose minimal media with appropriate supplements, or yeast peptone dextrose-rich medium (YPD) with added antibiotics [17]. *E. coli *was grown in NZCYM medium with antibiotics.

### Plasmid construction

Plasmid pGPch-1 (gift of Stefanie Pöggeler) [19] was the source of the *Pch *PRP8 intein and contains an N-terminal extein of glutathione S-transferase (GST) and a C-terminal extein of a 6 × His epitope tag. pRR01 is derived from pGPch-1. The ΔE sequence [9], 5'-GGCGCCGATGATTCGGCT-3', coding for GADDSA, was replaced with **5'-**GGCGCCGCAG GTGCAGGTGC AGGTGCAGCT AGCGCAGCAGGTACCGCGGG TGCTGGTGCT GGTGCTTCGG CT-3' (NheI and KpnI sites underlined), to code for the protein sequence GAAGAGAGAASAAGTAGAGAGASA. Six codons within the intein sequence were optimized for expression in yeast by site-directed mutagenesis using the QuikChange^® ^protocol (Stratagene, La Jolla, CA): Leu2 (CTC to TTG); Gly5 (GGG to GGA); Leu8 (CTC to TTG); Arg10 (CGA to AGA); Arg46 (CGC to AGA); and Arg49 (CGC to AGA). Numbering is relative to Cys (in the sequence CLAK) at position 1.

Plasmids pRR02-05 were derived from pRR1. The *G418^R ^*gene, the *Hyg^R ^*gene, the *his5^+ ^*gene of *Schizosaccharomyces pombe *and the *lexA-VP16 *fusion were PCR amplified from pBS7, pBS4, and pDH5 (YRC, University of Washington) [57] and pBS LexA::VP16-SV40 (gift of Tzumin Lee, Janelia Farm Research Campus) [58], respectively. The ORFs were amplified with NheI and KpnI ends and inserted at the NheI/KpnI junction in pRR01. All amplifications for cloning were carried out using Vent_R_^® ^DNA polymerase (New England Biolabs, Beverly, MA).

Plasmids pRR11-15 and pRR21-25 include the intein sequence from the corresponding marked inteins in the pRR02-05 series. However the N-extein is either GFP or GFP with a nuclear localization signal, NLS-GFP, and the C-extein is a FLAG^® ^epitope. In addition protein linker sequences have been inserted at the junctions between ORFs. These linkers, L2-L5 in Figure 4, encode the following protein sequences, with adjacent sequences in parentheses: L2, (...LYK)-GAGAGAGAGAG-(FWEKACLAK...); L3, (...KGA)-AGAGAGAA-(SG...); L4, AGAGAGA-(SAQ....); and L5, (SGFE)-GSG. The underlined sequences are from the N-and C-terminal exteins of *P. chrysogenum *Prp8.

As described in Table 1 these constructs were cloned into 3 base plasmids: pGEX-4T-1 (Amersham Bioscience, Europe GmbH, Freiburg, Germany); pET-Duet modified to contain only one promoter (EMD Biosciences, San Diego, CA), and pBM258 [59]. The complete DNA sequences are deposited in Genbank [60].

**Table 1:** Plasmids

Name	Base plasmid	Promoter	Origin/marker	Extein[intein]extein
pGPch-1	pGEX-4T-1	tac	ori/Amp	GST[*Pch *PRP8]HIS_6_
pRR01	pGEX-4T-1	tac	ori/Amp	GST[*Pch *PRP8 linker]HIS_6_
pRR02	pGEX-4T-1	tac	ori/Amp	GST[*Pch *PRP8 G418^R^]HIS_6_
pRR03	pGEX-4T-1	tac	ori/Amp	GST[*Pch *PRP8 HIS5]HIS_6_
pRR04	pGEX-4T-1	tac	ori/Amp	GST[*Pch *PRP8 HygB^R^]HIS_6_
pRR05	pGEX-4T-1	tac	ori/Amp	GST[*Pch *PRP8 LexA-VP16]HIS_6_
pRR11	pET	T7	ori/Amp	GFP[*Pch *PRP8 linker]FLAG
pRR12	pET	T7	ori/Amp	GFP[*Pch *PRP8 G418^R^]FLAG
pRR13	pET	T7	ori/Amp	GFP[*Pch *PRP8 HIS5]FLAG
pRR14	pET	T7	ori/Amp	GFP[*Pch *PRP8 HygB^R^]FLAG
pRR15	pET	T7	ori/Amp	GFP[*Pch *PRP8 LexA-VP16]FLAG
pRR21	pBM258	Gal	cen/ura3	NLS-GFP[*Pch *PRP8 linker]FLAG
pRR22	pBM258	Gal	cen/ura3	NLS-GFP[*Pch *PRP8 G418^R^]FLAG
pRR23	pBM258	Gal	cen/ura3	NLS-GFP[*Pch *PRP8 HIS5]FLAG
pRR24	pBM258	Gal	cen/ura3	NLS-GFP[*Pch *PRP8 HygB^R^]FLAG
pRR25	pBM258	Gal	cen/ura3	NLS-GFP[*Pch *PRP8 LexA-VP16]FLAG
pRR26	pBM258	Gal	cen/ura3	NLS-GFP (no intein)

All plasmids were constructed in this study except pGPch-1 [19]. In the table the promoter that drives expression of the extein-intein is indicated.

### Episomal expression of inteins in E. coli and yeast

Expression in *E. coli *was performed in Tuner™ (DE3) with pLysSRARE to express rare tRNAs. Bacteria were grown to early log phase in NZCYM medium supplemented with ampicillin (100 μg/ml) and chloramphenicol (34 μg/ml) then induced with 0.2 mM isopropyl β-D-1-thiogalactopyranoside (IPTG) for 17 h at 20°. Culture densities were measured with a Klett-Summerson Colorimeter.

For plasmid-based expression in yeast from the *GAL1 *promoter, the strain YL (2,4) LU was used with various plasmids based on pBM258. Cells were grown overnight at 30°in S-medium -ura + 2% raffinose. When cells reached a turbidity of 20 Klett units, galactose was added to a final concentration of 2% and growth was continued to 100 Klett units.

### Western blot analysis and the estimation of splicing efficiency

Equivalent amounts of cells were pelleted, precipitated with 10% TCA [61], resuspended in sample buffer and separated by SDS-PAGE. Three identical gels were prepared: one directly stained by Coomassie^® ^Brilliant Blue for detection of total protein and two transferred to Immobilon™ (Millipore, Billerica, MA) for Western blot analysis. Antibodies were either directed against GST (Santa Cruz Biotechnology, Santa Cruz, CA), Penta-His (Qiagen, Valencia, CA), GFP (Roche Applied Science, Indianapolis, IN) or the FLAG epitope (anti-FLAG^® ^M2, Sigma-Alrich, St. Louis, MO). Antibodies were detected using Alexa Fluor 680 anti-IgG secondary (Invitrogen, Carlsbad, CA) and visualized on an Odyssey Infrared Imaging System (LI-COR Biosciences, Lincoln, NE). The Odyssey software was used to quantify band intensity. To estimate the limits of detection for the anti-GFP antibody, Western blot analysis was performed on serial dilutions of extracts from strain YL (2,4) LU expressing NLS-GFP from plasmid pRR26.

### Genomic integration of marked inteins into the *CMD1 *locus

The intein cassettes were amplified from the pRR11-15 plasmids using the Expand Long Template PCR System (Roche, Indianapolis, IN). Primers included a 40 base extension homologous to the target *CMD1 *gene, a short linker sequence encoding L1 and L5 in Figure 4, and ~ 23 bp homologous to the ends of the intein sequence. The sequences of the primers were: 5'-TAGTGAATTTTTGGCTCTGATGTCTCGTCAACTCAAATCA GGTTCAGGT **AGTAAAGGAG AAGAACTTTT CACTG**-3' and 5'-ATACTTTAAAAGCTTCTAGTAGTTCTTGTTCAGAGTCATT TCCAGATCC **TTCGAAACCA CTGTTGTGCA G**-3' in which bold sequence is homologous to the intein cassette and underlined is homologous to the *CMD1 *site of insertion. The amplification and integration protocols are described at the Yeast Resource Center web site http://depts.washington.edu/yeastrc/pages/fm_3.html. KGY315 was the host except in the case of the transformation of the LexA-VP16-intein, which was transformed into YL [6,4] LU.

Fluorescence microscopy was performed on a DeltaVision^® ^imaging system (Applied Precision, Issaquah, WA) as previously reported [17]. Protein extraction and Western blot analysis were performed as described above using anti-GFP and anti-calmodulin antibodies [62]. The correct integration of the inteins was confirmed by four different PCR reactions using the genomic DNA as template and primer pairs from these regions: 5' UTR of *CMD1 *as forward primer (f.p.) × GFP as reverse primer (r.p.); GFP (f.p.) × C-terminal intein (r.p.); C-terminal intein (f.p.) × 3' UTR of *CMD1*(r.p.); and 5' UTR of *CMD1 *(f.p.) × 3' UTR of *CMD1*(r.p.).

## Authors' contributions

RR carried out the plasmid constructions, molecular genetic studies and Western blot analysis, drafted the manuscript and conceived of the project. LA carried out Western blot analysis, molecular genetic studies and fluorescence microscopy. TD helped to direct the study and draft the manuscript. EM co-ordinated and directed the study, carried out fluorescence microscopy and drafted the manuscript. All authors participated in the design of the study. All authors read and approved the final manuscript.
